# ABCA1 overexpression worsens colorectal cancer prognosis by facilitating tumour growth and caveolin‐1‐dependent invasiveness, and these effects can be ameliorated using the BET inhibitor apabetalone

**DOI:** 10.1002/1878-0261.12367

**Published:** 2018-09-17

**Authors:** Cristina Aguirre‐Portolés, Jaime Feliu, Guillermo Reglero, Ana Ramírez de Molina

**Affiliations:** ^1^ Molecular Oncology IMDEA Food Institute CEI UAM + CSIC Madrid Spain; ^2^ Medical Oncology La Paz University Hospital (IdiPAZ) CIBERONC, cátedra UAM‐AMGEN Madrid Spain

**Keywords:** ATP‐binding cassette transporter, apabetalone, colorectal cancer prognosis, reverse cholesterol transport

## Abstract

At the time of diagnosis, 20% of patients with colorectal cancer present metastasis. Among individuals with primary lesions, 50% of them will develop distant tumours with time. Therefore, early diagnosis and prediction of aggressiveness is crucial for therapy design and disease prognosis. Tumoral cells must undergo significant changes in energy metabolism to meet increased structural and energetic demands for cell proliferation, and metabolic alterations are considered to be a hallmark of cancer. Here, we present the ATP‐binding cassette transporter (ABCA1), a regulator of cholesterol transport, as a new marker for invasion and colorectal cancer survival. ABCA1 is significantly overexpressed in patients at advanced stages of colorectal cancer, and its overexpression confers proliferative advantages together with caveolin‐1 dependent‐increased migratory and invasive capacities. Thus, intracellular cholesterol imbalances mediated by ABCA1 overexpression may contribute to primary tumour growth and dissemination to distant locations. Furthermore, we demonstrate here that increased levels of apolipoprotein A1 (APOA1), a protein involved in cholesterol efflux and high‐density lipoprotein constitution, in the extracellular compartment modulates expression of ABCA1 by regulating COX‐2, and compensate for ABCA1‐dependent excessive export of cholesterol. APOA1 emerges as a new therapeutic option to inhibit the promotion of colorectal cancer to metastasis by modulating intracellular cholesterol metabolism. Furthermore, we propose apabetalone, an orally available small molecule that is currently being evaluated in clinical trials for the treatment of atherosclerosis, as a new putative therapeutic option to prevent colorectal cancer progression by increasing APOA1 expression and regulating reverse transport of cholesterol.

**Abbreviations**
ABCA1: ATP‐binding cassette transporter
APOA1: apolipoprotein A1
CAV‐1: caveolin‐1
CI: confidence interval
CMS: consensus molecular subtypes
COX‐2: cyclooxygenase 2
CRC: colorectal cancer
DMEM: Dulbecco′s modified Eagle medium
E‐cad: E‐cadherin
EMT: epithelial‐to‐mesenchymal transition
FA: fatty acids
hAPOA1: human recombinant RNA against apolipoprotein A1
HDL: high‐density lipoprotein
LN: lymph node
MβCD: methyl‐β‐cyclodextrin
NoORF: no open reading frame
RCT: reverse cholesterol transport
SI: stage I‐colorectal cancer
SII: stage II‐colorectal cancer
SIII‐CRC: stage III‐colorectal cancer
SIV: stage IV‐colorectal cancer
VIM: vimentin

## 1 Introduction

Cancer is the second leading cause of mortality and is responsible of one sixth of deaths worldwide. Among the global leading causes of cancer death and considering both sexes, colorectal cancer (CRC) occupies the fourth position after lung, liver and stomach cancer (WHO, 2012). Although the 5‐year survival after diagnosis when metastasis is already present has improved in the last decade, this improvement is still less than 3% (Steeg, 2016). In most cases, early detection allows tumours to be successfully removed by surgery and increases treatment efficiency. Thus, an early diagnosis and prediction of aggressiveness becomes of vital importance for therapy design and disease prognosis. During tumorigenesis, a cascade of alterations affecting the expression of oncogenes and tumour suppressors takes place in the body of the patients. Together with this, significant changes in energy metabolism are needed to respond to new requirements of tumoral cells to sustain increased structural, energetic and biosynthesis precursor demands for cell proliferation (Cantor and Sabatini, 2012). Although metabolic alterations encountered in tumours are nowadays considered a hallmark of cancer (Hanahan and Weinberg, 2011), knowledge of fatty acid (FA) synthesis and lipid metabolism alterations during tumorigenesis is still slight. ATP‐binding cassette transporter (ABCA1) is a transmembrane protein responsible for reverse cholesterol transport (RCT) from the inner cell to circulatory system (Oram and Lawn, 2001). By interacting with ABCA1, apolipoprotein A1 (APOA1) is able to recover the extracted cholesterol and synthethize high‐density lipoproteins (HDL) (Wang and Tall, 2003). Both the overexpression and the downregulation of ABCA1 have been associated with tumorigenesis (Bi *et al*., 2016; Chou *et al*., 2015; Hedditch *et al*., 2014; Lee *et al*., 2013; Smith and Land, 2012; Vargas *et al*., 2015). Importantly, this cholesterol transporter is one of the members of a lipid metabolism signature related to poor prognosis in patients diagnosed with stage II colorectal cancer (SII‐CRC). This signature, ColoLipidGene, encompasses the transcriptional activation of four metabolism‐related genes: *ACSL1*,* ABCA1*,* AGPAT1* and *SCD* (Vargas *et al*., 2015). A meta‐analysis performed in our laboratory with more than 1000 patients, demonstrated that *ABCA1* is the only member of the ColoLipidGene signature whose overexpression by itself represents a significant association with overall survival in CRC (Fernández *et al*., 2017). It is our aim to understand the importance of *ABCA1* and cholesterol transport regulation in CRC dissemination during advanced stages of the disease and its potential as a therapeutic target. BET inhibitors have been proposed as suppressors of tumorigenesis in several types of cancers (Cheng *et al*., 2013; Puissant *et al*., 2013; Shi *et al*., 2014); among these, apabetalone (RVX‐208; Resverlogix, Calgary, Canada; T3E6L1) is the most advanced in terms of clinical development. For the first time, we demonstrate here the potential of apabetalone as a regulator of ABCA1 and the reverse cholesterol transport. By promoting *APOA1* transcription, we propose here a new strategy based on apabetalone treatment to avoid CRC progression to invasive stages of cancer.

## 2 Materials and methods

### 2.1 Patients and samples

In all, 100 formalin‐fixed, paraffin‐embedded samples from La Paz University Hospital were obtained with the understanding and written consent of each patient and with the approval of the human research Ethics Review Committee of La Paz University Hospital (HULP‐PI‐1452). The methodologies in this study conformed to the standards set by the Declaration of Helsinki. Inclusion criteria were the following: age ≥18, completely resected rectal cancer or colon adenocarcinoma located at ≥15 cm from the anal verge as determined by endoscopy or above the peritoneal reflection in the surgical resection, confirmed stage II AJCC/UICC primary CRC and follow‐up of at least 36 months.

### 2.2 Generation of stable cell lines and drug treatments

DLD‐1 (CCL‐221™) and Caco‐2 (HTB‐37™) were purchased from the American Type Culture Collection (ATCC^®^, Manassas, VA, USA). Lentiviruses were produced in HEK293T cells. Selection was performed over 12 days by adding 2 μg·mL^−1^ puromycin and 3 μg·mL^−1^ blasticidin (Sigma‐Aldrich, Alcobendas, Spain) for no open reading frame (NoORF and *ABCA1* constructs, respectively. Methyl‐β‐cyclodextrin (MβCD) was purchased from Sigma (Cat. C4555). Human recombinant apolipoprotein A1 (hAPOA1) (Sigma‐Aldrich; Cat. SRP.4693) was used in a final concentration of 40 μg·mL^−1^. The absence of mycoplasma was confirmed by PCR every second week.

### 2.3 Three‐dimensional cell culture

A total of 10 000 cells were suspended in a mix of Dulbecco′s modified Eagle medium (DMEM; Lonza. Cultek S.L.U, Madrid, Spain), 10% FBS and Matrigel^®^ Growth Factor Reduced Basement Membrane Matrix (Corning^®^; Cat. 356230) (20% of cells and 80% of Matrigel^®^). Pictures were taken every 24 h using a Leica DM IL microscope and registered using las software (L'Hospitalet de Llobregat, Barcelona, Spain).

### 2.4 Cholesterol efflux assay

Intracellular cholesterol and cholesterol present in culture medium was measured using the Cholesterol Efflux Fluorometric Assay Kit (Cat. K582‐100; BioVision, Inc., Milpitas, CA, USA)

### 2.5 Cell proliferation assays

xCELLigence (ACEA Biosciences, Inc., Izasa Scientific, L'Hospitalet de Llobregat, Barcelona, Spain) was used to analyze cell proliferation. Ten thousand cells were seeded in 16‐well E‐plates (Cat. 05469830001), and a time‐lapse experiment was performed with the real‐time cell analysis instrument. Graphical representations were designed and statistically analyzed with graphpad prism software (La Jolla, CA, USA).

### 2.6 Migration and invasion assays

Crystal violet staining was performed in cells grown in Corning^®^ BioCoat™ Control Inserts 8 mm PET membrane (cat. 354578) and in a Corning^®^ BioCoat™ Matrigel^®^ invasion chamber (cat. 354480). Images were captured using an Olympus CKX41 microscope (Olympus Iberia S.A.U, L'Hospitalet de Llobregat, Barcelona, Spain) with 20× LCAch objective and getit software (Olympus) was used for the acquisition. Migration and invasion were quantified using imagej software (National Institutes of Health, Bethesda, MD, USA), and statistical analysis was performed with graphpad prism software.

### 2.7 Immunofluorescence and focal adhesion quantification

Cells were fixed in 4% paraformaldehyde‐PBS (Santa Cruz Biotechnology, CAS30525‐89‐4, Madrid, Spain) and blocked with 10% FBS. Primary antibodies were incubated overnight. After 45 min at room temperature, secondary antibodies (Alexa 488, Alexa 594; 1 : 1000) were harvested and cells were rinsed with PBS‐Triton 0.03%. Images were captured using a Leica DM IL microscope and registered using las software. For the quantification of FA sizes, the protocol previously published by Utku Horzum and collaborators was followed (Horzum *et al*., 2014). The localization of *ABCA1* and α‐sodium potassium ATPase was studied with a Leica SP5 Confocal Microscope.

### 2.8 Biochemical analysis and quantitative PCR

For immunoblotting, cells were lysed with Laemmli buffer. Measurements of APOA1 concentration were done by ELISA following the recommendations of the supplier (Abcam, Cambridge, UK; ab108804). RNA was extracted using Tri Reagent (Sigma‐Aldrich). Quantitative real‐time PCR was performed in the QuantStudio 12K Flex System Real‐Time PCR System (Life Technologies, Alcobendas, Spain). Values were corrected by GAPDH expression. The 2^−∆∆Ct^ method was applied to calculate the relative gene expression.

### 2.9 Antibodies

The following antibodies were used: mouse monoclonal ABCA1 antibody (AB.H10; Abcam. Cat. ab18180; Abcam); anti‐apolipoprotein AI antibody (EP1368Y; Abcam. Cat. ab52945); rabbit polyclonal caveolin‐1 antibody (Cat. Abcam. ab2910); monoclonal anti‐alpha‐tubulin, clone DM1A (Sigma‐Aldrich. Cat. T9026); purified mouse anti‐E‐cadherin (BD Biosciences. Cat. 610182, San Agustín de Guadalix, Madrid, Spain); mouse monoclonal anti‐vimentin (Progen. Cat. 61013, Heidelberg, Germany); rabbit polyclonal phospho‐histone H3 (Ser10) (Cell Signaling. Cat. D2C8, L 'Hospitalet de Llobregat, Barcelona, Spain); mouse monoclonal RHOA antibody (1B12; Abcam. Cat. 54835); mouse monoclonal Anti‐alpha 1 Sodium Potassium ATPase antibody [464.6]; Abcam. Cat. ab7671.

### 2.10 Statistical analysis

The significance of the differences between groups was determined by *t*‐test. All reported *P*‐values were two‐sided. The statistical analysis was performed using graphpad prism software: n.s. *P* > 0.05; *P < 0.05; **P < 0.01; ***P < 0.001; ****P < 0.0001.

## 3 Results

### 3.1 *ABCA1* as a putative marker for colorectal cancer malignancy

**Figure 1:**
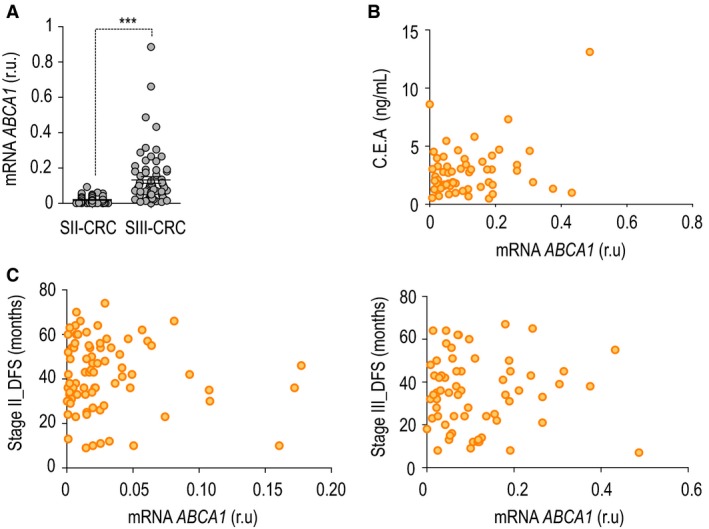
ABCA1, a new putative marker for CRC prognosis. (A) ABCA1 mRNA expression in SII‐CRC and SIII‐CRC patients. (B) Correlation between ABCA1 expression and carcinoembryonic antigen. (C) ABCA1 mRNA levels of expression correlated with disease‐free survival (DFS) in SII‐CRC (left‐hand panel) and SIII‐CRC patients (right‐hand panel).

Stage II of colorectal cancer is characterized by invasion of the muscularis propria and/or subserosa, non‐peritonealized pericolic or perirectal tissue, with no invasion of lymph nodes. In contrast, stage III patients (SIII‐CRC) always display at least one lymph node affected with no distant metastasis. To evaluate the importance of *ABCA1* regulation in CRC staging, we analyzed mRNA expression in 71 SII‐CRC and 66 SIII‐CRC patients (University Hospital La Paz, Madrid). Expression of *ABCA1* mRNA was assessed by quantitative real‐time PCR, and we confirmed a significant increase in SIII‐CRC patients (Fig. 1A). Together with other markers, the carcinoembryonic antigen is used in the surveillance of CRC patients as a clinical parameter for prognosis determination (Duffy, 2001; Goldstein and Mitchell, 2005; Thomson *et al*., 1969). In our patient cohorts, we found a weak positive correlation between *ABCA1* and pre‐operatory circulatory levels of carcinoembryonic antigen (Fig. 1B). No correlation with disease‐free survival was found in either SII‐CRC or SIII‐CRC patients (Fig. 1C). When other clinical features were analyzed, we found no correlation with *ABCA1* levels (Table 1). In a previous work published by our laboratory, the authors performed a meta‐analysis to determine whether *ABCA1* gene expression was associated with death in 1073 CRC patients at all the stages of the disease [from stage I CRC (SI‐CRC) to stage IV CRC (SIV‐CRC)]. Notably, *ABCA1* expression had a risk effect on overall survival, and the pooled Hazard ratio (HR) was 1.29 [95% confidence interval (CI) 0.99–1.69]. In the same direction, expression of the cholesterol transporter increased risk of recurrence in patients from stages I to III [HR = 1.86 (95% CI 1.37–2.53) (Fernández *et al*., 2017)]. These data, together with the results that we present here, allow us to postulate *ABCA1* as a new putative marker whose levels of expression could be used as a prognostic biomarker in CRC patients.

**Table 1:** Levels of ABCA1 mRNA are represented as the mean of all the patients together with the standard error of the mean. Statistical significance is shown for each stage after comparing parameters. The comparison is made between less malignant and more malignant situations

Clinical features	ABCA1 mRNA	*P*‐value
TNM stage		
Stage II	0.01995 ± 0.002115	<0.0001
Stage III	0.1326 ± 0.01892	
Primary tumour (T)		
T2/3	0.1308 ± 0.01640	0.1556
T4	0.09311 ± 0.02623	
Affected LN		
0‐3	0.1053 ± 0.01495	0.2940
≥4	0.1176 ± 0.02080	
Perforation		
Negative	0.1171 ± 0.01507	0.7321
Positive	0.1056 ± 0.02956	
Vascular invasion		
Negative	0.1134 ± 0.01526	0.8897
Positive	0.1173 ± 0.01526	
Lymphatic invasion		
Negative	0.1033 ± 0.01483	0.2703
Positive	0.1338 ± 0.02551	

### 3.2 *ABCA1* overexpression confers proliferative advantages, promotes epithelial‐to‐mesenchymal transition (EMT) and leads to increased invasiveness

**Figure 2:**
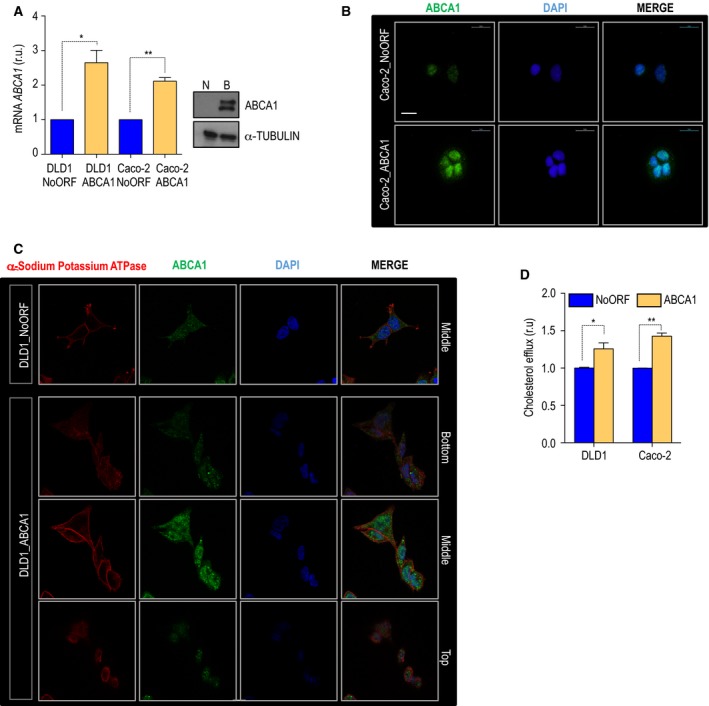
ABCA1 overexpression in two human CRC‐derived cell lines. (A) mRNA and ABCA1 protein levels in DLD1 and Caco‐2 cells transduced for ABCA1 overexpression. Control cell line was transduced with an empty vector containing a DNA fragment with no open reading frame (NoORF). The values in the histograms correspond to mean ± SEM. DLD1_NoORF: 1.000 ± 3.576e‐007; DLD1_ABCA1: 2.649 ± 0.3553. Caco‐2_NoORF: 1.000 ± 2.146e‐006, Caco‐2_ABCA1: 2.115 ± 0.1029. The significance of the analysis was determined by *t*‐test. (B) ABCA1 localization in Caco‐2 control and Caco‐2_ABCA1. DNA was stained with 4′,6‐diamino‐2‐phenylindole (DAPI) (blue); ABCA1 in green. Scale bar corresponds to 20 μm. (C) Localization of overexpressed ABCA1 in DLD1 cells. α‐Sodium potassium ATPase was used to stain the plasma membrane (in red). ABCA1 is shown in green. DNA was stained with DAPI (blue). Three sections are shown: the bottom part of the cell corresponds to the first row, then the middle portion of the cells and in the third row, the upper part of the cell is included. (D) Cholesterol efflux in DLD1 and Caco‐2 stable cell lines. *n* = 3; for each experiment every condition was analyzed in triplicate. The values in the histograms correspond to mean ± SEM. Significance between groups was determined by *t*‐test. DLD1_NoORF: 1.129 ± 0.1289; DLD1_ABCA1: 1.213 ± 0.2134. Caco‐2_NoORF: 1.000 ± 1.654e‐005; Caco‐2_ABCA1: 1.342 ± 0.08448.

**Figure 3:**
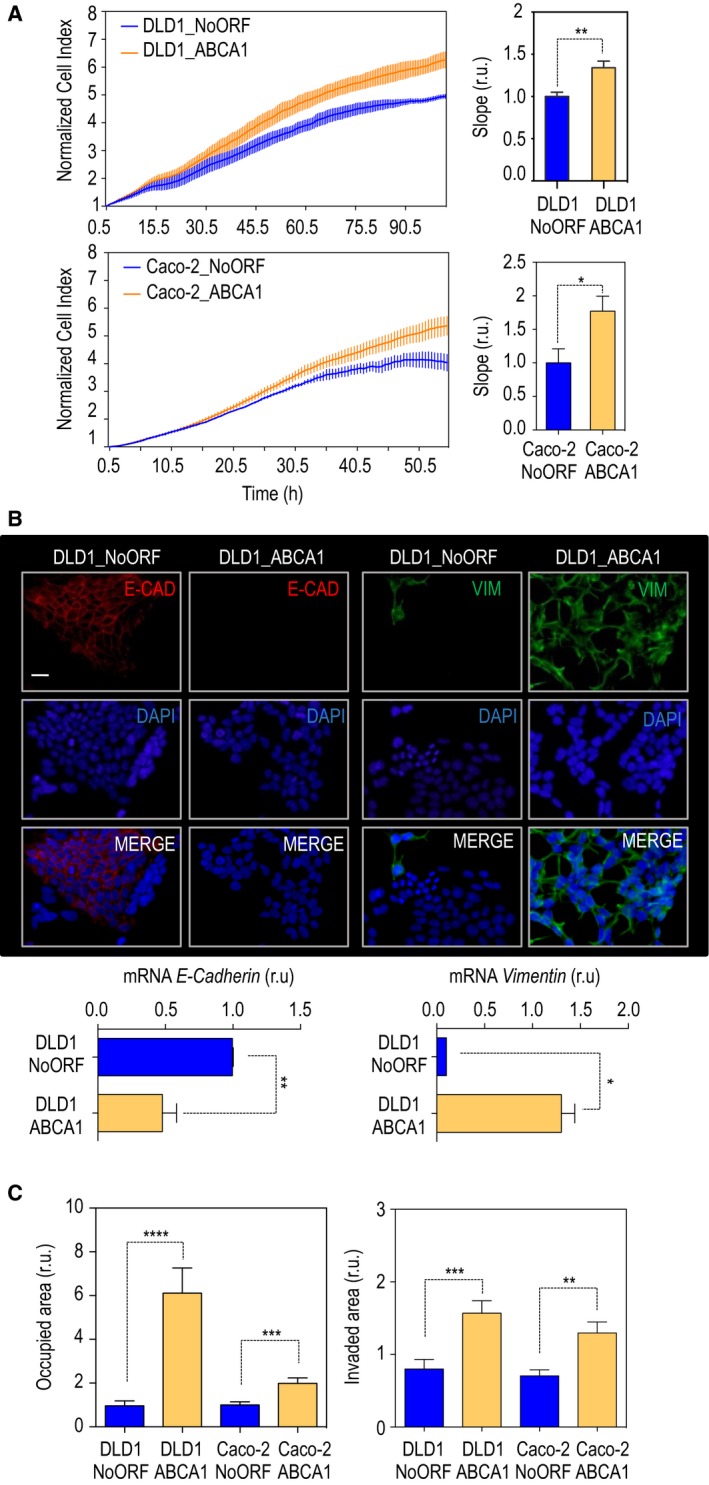
ABCA1 overexpression gives proliferative advantages, promotes EMT and favours invasion. (A) Cell proliferation assay in cells overexpressing ABCA1. *n* = 3. The values in the histograms correspond to mean ± SEM. The significance of differences between experimental groups was determined by *t*‐test. (B) Protein localization and mRNA levels of expression of two well established markers for EMT, E‐cad (red) and Vim (green). DNA is shown in blue (DAPI). N = DLD1_NoOF; B = DLD1_ABCA1. Scale bar corresponds to 20 μm. The values in the histograms correspond to mean ± SEM. The significance of the differences between experimental groups was determined by *t*‐test. (C) Quantification of cell migration (left‐hand panel) and cell invasion (right‐hand panel) in DLD1 and Caco‐2 cells overexpressing ABCA1 and compared with control cells. *n* = 3. For each experiment, every condition was analyzed in duplicate; six fields per condition were quantified. Mean ± SEM is represented in the histograms. The significance between groups was determine by *t*‐test analysis.

To decipher the molecular mechanisms behind the association between the expression of the cholesterol transporter and CRC prognosis, two stable cells lines, Caco‐2 and DLD1, were generated. Basal levels of ABCA1 in Caco‐2 are significantly higher than those displayed by DLD1 cells (Fig. S1A); however, levels of expression of the APOA1 are also increased when we compare Caco‐2 with DLD1 cells (Fig. S1A). DLD1 and Caco‐2 cells were transduced with exogenous human ABCA1 (Figs 2A and S1B). First, the localization of the protein was analyzed by immunofluorescence. In Caco‐2 cells, the endogenous protein was detectable in the nucleus of control cells. Upon overexpression, levels of ABCA1 were increased in the nuclear compartment and were detectable in cytoplasmic vesicles (Fig. 2B). Although in DLD1 cells that overexpressed ABCA1 the localization was mainly cytoplasmic, the protein was also detectable at the plasma membrane and at the nucleus (Fig. 2C). High levels of the transporter led to a significant increase in APOA1‐dependent cholesterol efflux, demonstrating the functionality of exogenous ABCA1 (Fig. 2D). Next, proliferation and cell viability were monitored in a real‐time experiment applying xCELLigence technology. Overexpression of *ABCA1* resulted in a significant increase in cell proliferation (Fig. 3A). Together with this, 3.3% of DLD1_NoORF cells were positive for histone H3 phosphorylation (3.244 ± 0.53119), whereas up to 5.4% of DLD1_ABCA1 displayed positive staining (5.400 ± 0.5669). An increased percentage of proliferating cells was also found in Caco‐2_ABCA1 cells (Fig. S1C). To achieve malignancy, cells need to downregulate a battery of genes as well as activate the expression of some others in a process called EMT (Kalluri and Weinberg, 2009). We analyzed the levels of E‐cadherin (E‐CAD) and vimentin (VIM), two classical markers of EMT. Both protein and mRNA levels of E‐cad were significantly lower in DLD1_ABCA1, whereas there was a significant increase in Vim levels (Fig. 3B). When Caco‐2 cells were monitored for mRNA levels of these markers, we observed a significant decrease in E‐cad in parallel with a mislocalization of the protein. Together with this, a significant decrease in the levels of Vim was present in Caco‐2_ABCA1 cells (Fig. S1D). Performing classical transwell assays, we observed a six‐fold increase in DLD1_ABCA1 migration capacity compared with control cells (Fig. 3C; left‐hand panel). When Caco‐2 cells were monitored, a highly significant increase was also observed (Fig. 3C). Importantly, when the same experiment was performed using transwells coated with collagen, we confirmed an increased invasiveness in both cell lines overexpressing *ABCA1* (Fig. 3B; right‐hand panel). Thus, ABCA1 could favour tumorigenesis by promoting the growth of the primary tumour, by increasing proliferation and the formation of distant lesions, by inducing EMT and by the subsequent mobilization of tumour cells.

### 3.3 *ABCA1* overexpression favours spheroid formation and promotes invasion in a three‐dimensional *in vitro* model

**Figure 4:**
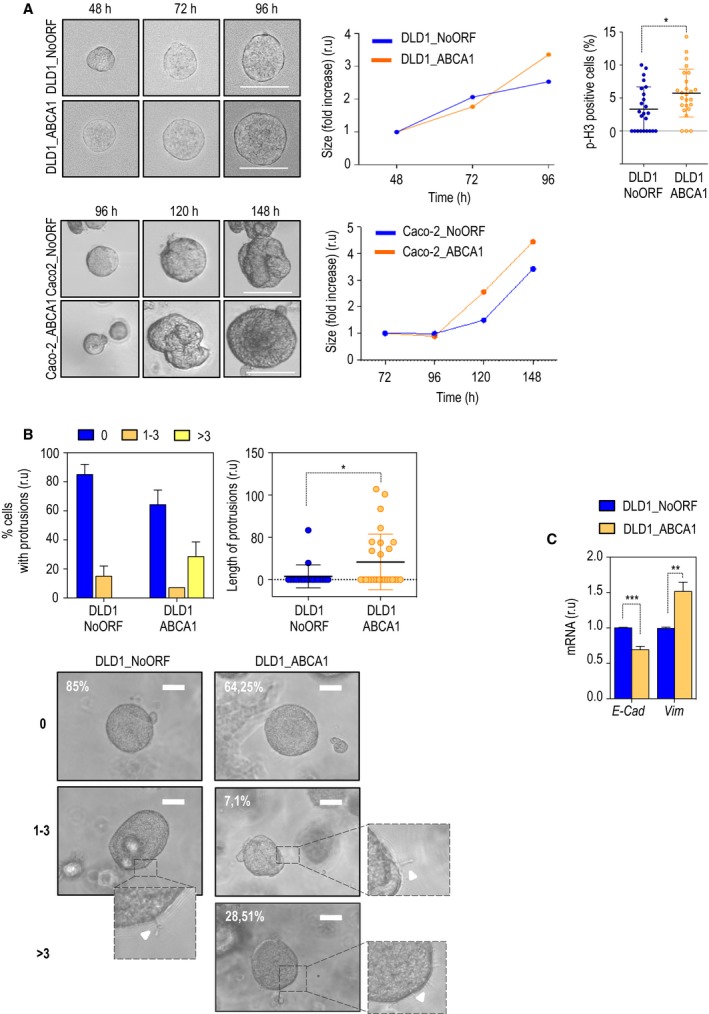
ABCA1 overexpression favours spheroid formations and promotes invasion in a three‐dimensional model. (A) Representative images of cell growth in a spheroid formation assay in basement membrane matrices (Matrigel™). Scale bar corresponds to 50 μm. Growth of DLD1 spheres is represented in the middle panel. The graph presents the average of 30 different spheres. The percentages of cells that were positive for histone H‐3 phosphorylation are presented in the dot plot graph. n = 3; 10 pictures per condition were taken and quantified. The values in the histograms correspond to mean ± SEM. The significance of differences between experimental groups was determined by *t*‐test. DLD1_NoORF: 3.311 ± 0.6882; DLD1_ABCA1: 5.736 ± 0.7247. (B) The percentage of spheroids that present protrusions is shown in the left‐hand panel. The values in the histograms correspond to mean ± SEM. ANOVA analysis was performed to determine the significance of differences between groups (*F*_(4,9)_ = 6.146; *P* = 0.0115). In the dot plot graph, quantification of the longest protrusions per spheroid are represented. A *t*‐test analysis was used to determine the significance of the analysis. DLD1_NoORF: 3.914 ± 3.037; DLD1_ABCA1: 20.92 ± 6.231. The experiment was performed three times; 10–20 spheroids were quantified. Scale bars correspond to 100 μm. (C) Levels of expression of E‐cad and Vim mRNA. The values in the histograms correspond to mean ± SEM. The significance of differences between experimental groups was determined by *t*‐test. For E‐cad, DLD1_NoORF: 1.003 ± 0.003161; DLD1_ABCA1: 0.6925 ± 0.02835. For Vim, DLD1_NoORF: 0.9942 ± 0.009214; DLD1_ABCA1: 1.515 ± 0.07528.

To monitor cell malignancy in a system that better represents *in vivo* conditions, we developed spheroids derived from our stable cell lines. Single cells overexpressing ABCA1 (Fig. S2A) were embedded in basement membrane matrices containing laminin, collagen IV, heparin sulfate proteoglycans, entactin/nidogen, and a number of growth factors (Matrigel™). First, we observed a growth advantage in both DLD1 and Caco‐2 overexpressing *ABCA1* when compared with control cells, together with an increase in the mitotic population (Figs 4A and S2B)**.** Next, we interrogated our spheroids for invasiveness. Cells need to develop invadopodia and degrade the extracellular matrix during invasion. Thus, to understand whether ABCA1 overexpression leads to increased invasion in Matrigel™, we analyzed the number and the length of the invadopodia present in each sphere. The number of protrusions per spheroid was higher in those spheroids derived from DLD1_ABCA1 (Fig. 4B; left‐hand panel). By measuring the longest protrusion in each spheroid, we demonstrated that the length was significantly increased in cells overexpressing the transporter (Fig. 4B; right‐hand panel). When Caco‐2 were monitored, the overexpression of ABCA1 promoted the development of protrusions, whereas no significant differences were found when the length was measured (Fig. S2C). Together with this, statistically significant differences were found in *E‐cad* and *Vim* levels of expression (Fig. 4C). Together, these data support our previous results obtained in two‐dimensional cultures, suggesting that overexpression of *ABCA1* could be associated with increased invasive capacities in colorectal cancer‐derived cells.

### 3.4 Increased malignancy of CRC cells overexpressing *ABCA1* is dependent on caveolin‐1 (CAV‐1) and cholesterol transport

**Figure 5:**
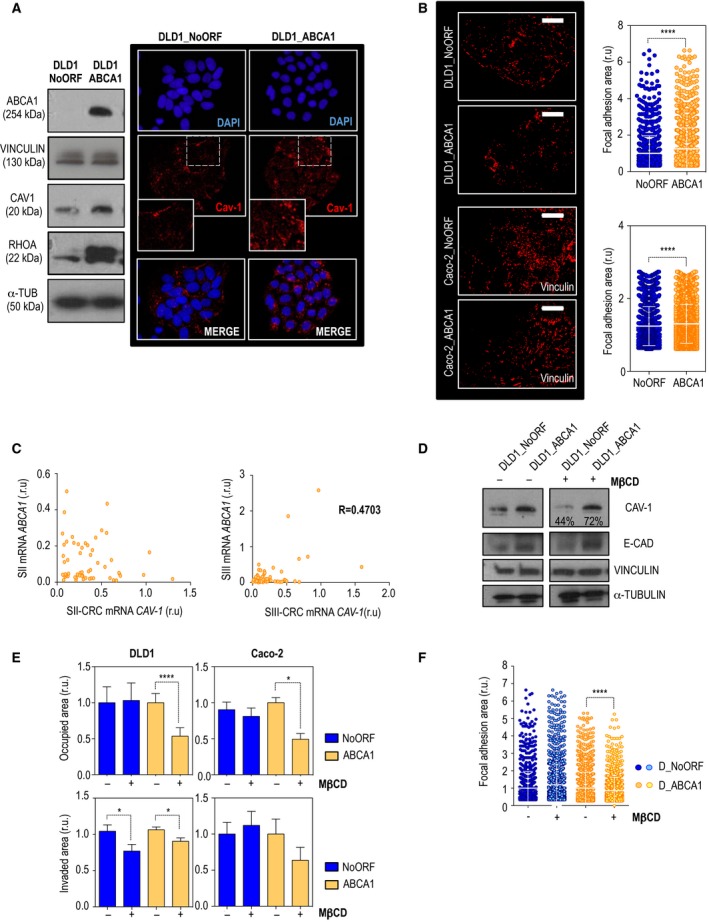
ABCA1 overexpression stabilizes CAV‐1, affecting the dynamics of cellular focal adhesions. Malignant phenotypes driven by ABCA1 were reversed by APOA1. (A) ABCA1, CAV‐1 and RHOA protein levels in DLD1 stable cell lines. Caveolae staining by CAV‐1 immunoblotting is shown on the left‐hand panel. DNA is shown in blue; CAV‐1 is shown in red. (B) FA areas were measured by vinculin immunostaining. Both DLD1 (upper panel) and Caco‐2 cell lines (lower panel) are shown. Scale bars correspond to 20 μm; r.u., relative units. The experiment was performed three times. For each experiment, 10 pictures were taken and the number of FA was normalized against the number of cells present in the field. The error bars in the histograms correspond to SEM. The significance of the differences between groups was determined by *t*‐test analysis. DLD1_NoORF: 1.000 ± 0.02838; DLD1_ABCA1: 1.252 ± 0.03057. Caco‐2_NoORF: 1.244 ± 0.01248, Caco‐2_ABCA1: 1.305 ± 0.007392. (C) Correlation between CAV‐1 and ABCA1 mRNA expression in SII‐ and SIII‐CRC patients. (D) Protein levels upon MβCD (4 mm) treatment in DLD1 cells. (E) Migration and invasiveness under MβCD treatment assessed in both DLD1 (left‐hand histograms) and Caco‐2 (right‐hand histograms) cell lines. *n* = 3. For each experiment, every condition was analyzed in duplicate; six fields per transwell were quantified. The error bars in the histograms correspond to SEM. The significance of differences between groups was determined by *t*‐test analysis. In the migration analysis with DLD1 cells, the following results were statistically significant: DLD1_ABCA1 control: 1.000 ± 0.07007, DLD1_ABCA1 + MβCD: 0.4931 ± 0.07956. For Caco‐2 cells: Caco‐2_ABCA1 control: 1.000 ± 0.1276; Caco‐2_ABCA1 + MβCD: 0.5360 ± 0.1169. Analysis of invasion in DLD1 cells: DLD1_NoORF control: 1.041 ± 0.08831, DLD1_NoORF+MβCD: 0.7689 ± 0.09205; DLD1_ABCA1 control: 1.063 ± 0.03943; DLD1_ABCA1 + MβCD: 0.9050 ± 0.04589. (F) Analysis of focal adhesion size using vinculin detection and quantification. Cells treated with the vehicle and with MβCD (4 mm) cells are shown. The experiment was performed three times. For each experiment, 10 pictures were taken and the number of FA was normalized against the number of cells present in the field. Mean ± SEM. The significance of differences between groups was determined by *t*‐test analysis. DLD1_ABCA1 control cells: 1.000 ± 0.02838; DLD1_ABCA1 + MβCD: 1.192 ± 0.03278.

Caveolae were proposed to be part of the complex regulating cholesterol efflux and to be the principal location at which plasma membrane cholesterol is exchanged between HDL and cells membrane (Luo *et al*., 2010; Qin *et al*., 2016). Increased transcription of *ABCA1* was associated with CAV‐1 stabilization in prostate cancer‐derived cells (Cohen *et al*., 2004; Her *et al*., 2013). We confirmed that CAV‐1 protein levels were higher in cells overexpressing the cholesterol transporter, whereas mRNA levels were comparable to control cells (Figs 5A and S3A). Together with this, an increase in caveolae formation was observed by immunodetection in DLD1_ABCA1 cells, as shown in the right‐hand panels of Fig. 5A. By RHOA activation, CAV‐1 is implicated in focal adhesion stability and turnover (Goetz *et al*., 2008; Grande‐García *et al*., 2007). In our system, *ABCA1* not only stabilized CAV‐1 but also caused an increase in total RHOA protein levels (Fig. 5A). CAV‐1 is already known to be implicated in migration by increasing focal adhesion turnover, polarization, velocity, persistence and directionality (Urra *et al*., 2012). Thus, we wondered whether ABCA1 was able to regulate focal adhesion formation through stabilization of CAV‐1. By vinculin immunodetection, we analyzed the status of focal adhesions in our model. Cells overexpressing *ABCA1* displayed a non‐significant increase in the number of focal adhesions (Fig. S3B). Notably, in DLD1_ABCA1 and Caco‐2_ABCA1 cell lines, vinculin‐dependent adhesion sites became larger, with an average fold increase of 1.228 ± 0.0323 (Fig. 5B, upper panel) and 1.244 ± 0.01248, respectively (Fig. 5B, lower panel). When patient samples were analyzed, no correlation between *ABCA1* and *CAV‐1* expression was observed in SII‐CRC (Fig. 5C). Importantly, a moderate positive correlation between the expression of both mRNAs was observed in SIII‐CRC (Fig. 5C). Thus, ABCA1 overexpression might lead to a stabilization of CAV‐1 protein in patients with colorectal cancer, leading to increased invasive capacities of tumour cells. To further confirm the dependence of CAV‐1 regulation in cell malignancy, we used the protein inhibitor MβCD to disrupt caveolae and repress *CAV‐1* expression**.** Addition of the drug in DLD1_NoORF produced a reduction in 56% of CAV‐1 protein levels. This reduction was compromised when DLD1_ABCA1 were treated (72% of CAV‐1) (Fig. 5D). Neither control or ABCA1‐overexpressing cells displayed any significant differences in proliferation when treated with MβCD (data not shown). MβCD treatment did not affect migration in control cells; however, the drug significantly reduced DLD1_ABCA1 and Caco‐2_ABCA1 migrating capacities (Fig. 5E). For DLD1 cells, we also confirmed a treatment‐dependent significant decrease in the invasive phenotype. Whereas no significant rescue was found in Caco‐2 control cells, a decreasing tendency was found in Caco‐2_ABCA1 (Fig. 5E). Together with these phenotypes, inhibition of CAV‐1 provoked a highly significant decrease in the size of focal adhesions (Fig. 5F). Thus, malignant phenotypes derived from ABCA1 overexpression are dependent on CAV‐1 stability and its role as a focal adhesion regulator.

### 3.5 APOA1 overexpression or exogenous human recombinant APOA1 inhibit cyclooxygenase 2 (*COX‐2*) expression and decrease malignant properties driven by ABCA1 overexpression

**Figure 6:**
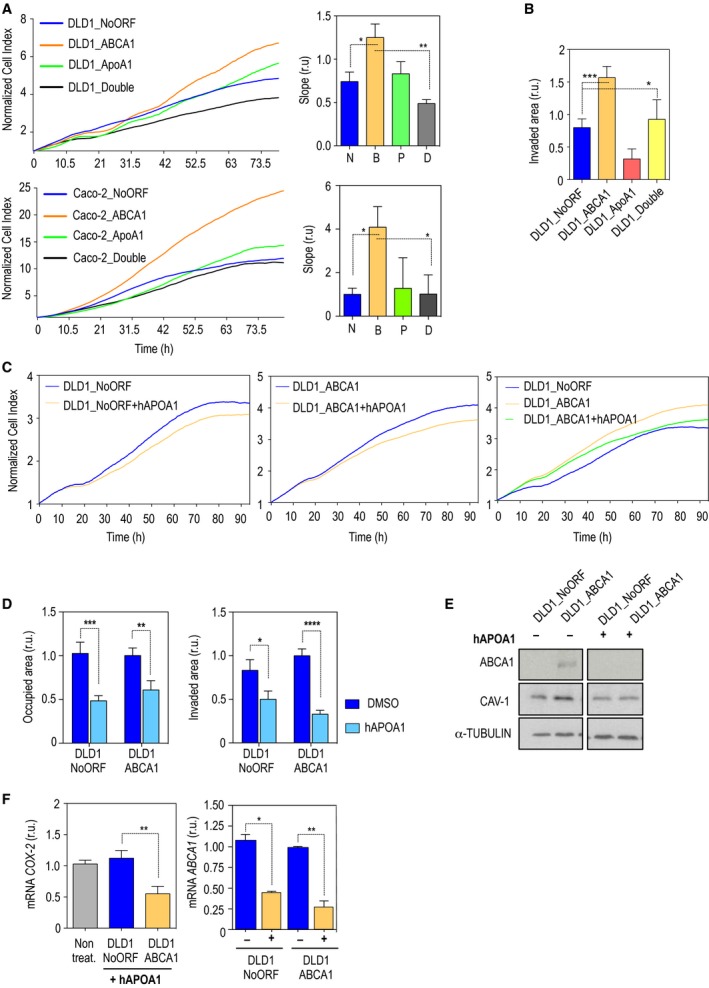
The presence of endogenous and exogenous APOA1 indicated a reduction in cell proliferation and decreased invasiveness by ABCA1 downregulation and CAV‐1 reduction. (A) Cell proliferation of DLD1 and Caco‐2 cells overexpressing ABCA1 and APOA1 alone or in the same population (DLD1_Double). The values in the histograms correspond to mean ± SEM. The significance of differences between experimental groups was determined by *t*‐test. (B) Matrigel™‐coated transwell analysis for invasiveness relative to control DLD1_NoORF cells. The values in the histograms correspond to mean ± SEM. The significance of differences between experimental groups was determined by *t*‐test. (C) Proliferation rate plots under hAPOA1 treatment. (D) Migration and invasion assays in cells treated with human recombinant APOA1. *n* = 3. For each experiment, every condition was analyzed in duplicate; six fields per transwell were quantified. The values in the histograms correspond to mean ± SEM. The significance of differences between experimental groups was determined by *t*‐test. DLD1_NoORF+DMSO: 1.026 ± 0.1288; DLD1_NoORF+hAPOA1: 0.4850 ± 0.05796. DLD1_ABCA1 + DMSO: 1.002 ± 0.08675; DLD1_ABCA1 + hAPOA: 0.6076 ± 0.1068. (E) Western blot for ABCA1 and CAV‐1 detection in cells treated and not treated with hAPOA1 (80 μg·mL^−1^). (F) Quantitative real‐time PCR for the detection of COX‐2 mRNA in the presence of hAPOA1. In the histogram on the right, the levels of ABCA1 mRNA expression are shown for control and DLD1_ABCA1 cells. The PCR was performed in triplicate with different cell stocks. Each sample was analyzed in triplicate. The values in the histograms correspond to mean ± SEM. The significance of differences between experimental groups was determined by *t*‐test. For migration, DLD1_NoORF + hAPOA1: 1.123 ± 0.07083. DLD1_ABCA1 + hAPOA1: 0.5527 ± 0.06850. For invasion, DLD1_NoORF + hAPOA1: 0.8321 ± 0.1226; DLD1_ABCA1 + hAPOA1: 0.5001 ± 0.09493.

Considering the essential contribution of *APOA1* in RCT, we investigated the role of this apolipoprotein in processing the excess of cholesterol and reversing the malignant phenotype driven by ABCA1 overexpression. Two additional stable cell lines were generated to overexpress either *APOA1* or both genes *ABCA1* and *APOA1* in the same population. First, when cell proliferation was analyzed, APOA1‐overexpressing cells showed proliferation rates that were comparable to those of the control (Fig. 6A). When both genes were upregulated, the high proliferation displayed under ABCA1 overexpression was reversed by the presence of high levels of APOA1 (Fig. 6A). In the same direction, overexpression of APOA1 was able to reduce the basal levels of invasion present in DLD1 control cells and ABCA1 overexpression phenotype was partially rescued in the presence of the APOA1 (Fig. 6B). To determine whether *APOA1* in the extracellular compartment would be able to compensate for the malignant features derived from ABCA1 overexpression, we used human recombinant APOA1 (hAPOA1) and monitored cell proliferation, migration and invasive capacities. First, hAPOA1 was able to reduce the proliferation rate of DLD1_ABCA1 and Caco‐2ABCA1 cells at levels that were comparable to those of the control (Figs 6C and S4). Together with this, a significant decrease in cell migration was observed upon hAPOA1 treatment (Fig. 6D). Furthermore, hAPOA1 compromised the invasiveness of both DLD1_NoORF and DLD1_ABCA1 cell lines (Fig. 6D). When the levels of CAV‐1 were monitored by Western blot, a clear reduction in its levels was detected upon treatment with hAPOA1 (Fig. 6E), accompanied by a reduction in ABCA1 protein levels (Fig. 6E). APOA1 overexpression has been associated with the inhibition of *COX‐2* expression in hepatocytes (Mao *et al*., 2014); as *COX‐2* inhibition is known to be able to downregulate both ABCA1 mRNA and protein (Chan *et al*., 2007), we analyzed the levels of the COX‐2 in our model. The addition of hAPOA1 in DLD1_ABCA1 cells gave rise to a significant decrease in *COX‐2* mRNA levels, accompanied by a significant decrease in ABCA1 (Fig. 6F). Thus, APOA1 overexpression reverses ABCA1‐derived malignant features not only by direct regulation of exported cholesterol but also by downregulating *COX‐2*, therefore reducing ABCA1 protein levels.

### 3.6 Apabetalone as a putative treatment for invasive CRC

**Figure 7:**
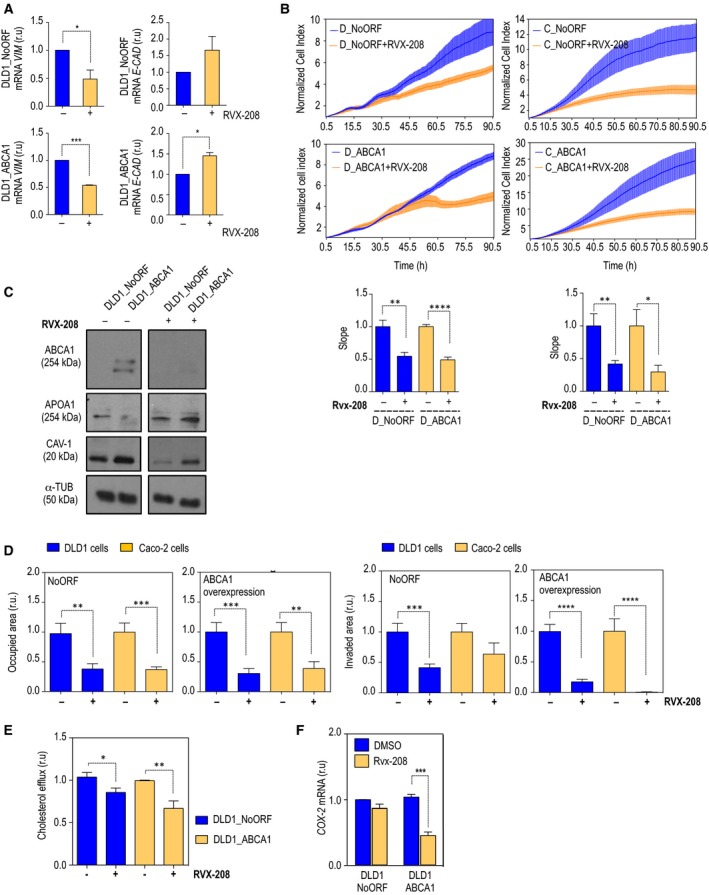
Treatment with apabetalone reverses the malignancy driven by ABCA1. (A) Levels of expression of EMT markers, Vim (left‐hand panels) and E‐cad (right‐hand panels). Cells were treated with RVX‐208 (40 μm) or DMSO as control. The values in the histograms correspond to mean ± SEM. The significance of differences between experimental groups was determined by *t*‐test. Each analysis was performed by triplicate. (B) Growth curve analysis and quantification. Both vehicle‐treated and RVX‐208‐treated cells were plotted. *n* = 3. D_NoORF = DLD1_NoORF; D_ABCA1 = DLD1_ABCA1; C_NoORF = Caco‐2_NoORF; C_ABCA1 = Caco2_ABCA1 cells. The values in the histograms correspond to mean ± SEM. The significance of differences between experimental groups was determined by *t*‐test. (C) ABCA1, APOA1 and CAV‐1 protein levels upon RVX‐208 treatment; α‐tubulin was used as loading control. (D) Quantification of migration and invasion assays, both in DLD1 (black) or Caco‐2 (grey) control cells and cells overexpressing ABCA1. *n* = 3. For each experiment, every condition was analyzed in duplicate; six fields per transwell were quantified. (E) Cholesterol efflux analysis in DLD1 control (black) cells and cells overexpressing ABCA1 (grey). The experiment was performed in triplicate, and for each repetition, every condition was analyzed in triplicate. (F) mRNA levels of *COX‐2* together with immunodetection of ABCA1 and CAV‐1 by Western blot. Vinculin was used as loading control.

**Figure 8:**
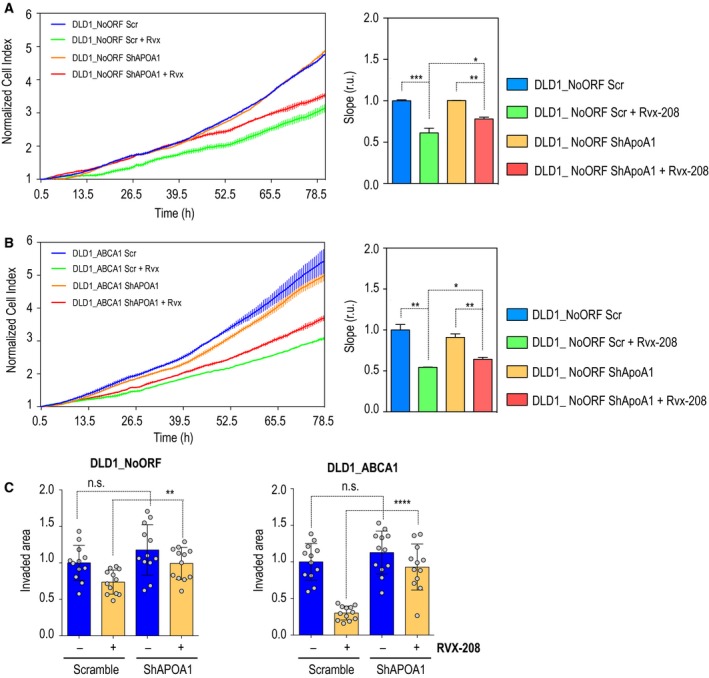
APOA1 silencing reversed the phenotype promoted by RVX‐208. (A) Cell proliferation assay in control cells treated with DMSO and RVX‐208 (80 μm). Scr, scrambled shRNA; shApoA1, short hairpin RNA against *APOA1* expression. The histogram represents the slopes of the curves. DLD1_NoORF transfected with shApoA1 and DMSO; in red, DLD1_NoORF transfected with shAPOA1 and treated with RVX‐208. The values in the histograms correspond to mean ± SEM. Significance between experimental groups were determined by *t*‐test. (B) Cell proliferation assay in ABCA1 overexpressing cells treated with DMSO and RVX‐208. Scr: scrambled short hairpin RNA; shAPOA1: short hairpin RNA against *APOA1* expression. The histogram represents the slopes of the curves. (C) Invasion assay performed in DLD1 cells. Left panel represents DLD1_NoORF data; right‐hand panel corresponds to DLD1_ABCA1. *n* = 3. For each experiment, every condition was analyzed in duplicate; six fields per transwell were quantified.

In 2010, Resverlogix developed a new molecule, apabetalone (RVX‐208), a BET inhibitor originally discovered in a screening for *APOA1* mRNA inducers in cultured hepatocytes (Gilham *et al*., 2016; McNeill, 2010). RVX‐208 is the most advanced BET inhibitor in clinical development. While this manuscript was in preparation, apabetalone was entered in a Phase 3 clinical trial for high‐risk cardiovascular disease (CVD) patients with type 2 diabetes mellitus and low HDL (BETonMACE). Therefore, considering that RVX‐208 could mimic hAPOA1 effect and to test whether it could be included as a complementary treatment for CRC patients, we analyzed whether ABCA1‐dependent malignant features could be ameliorated by the RVX‐208 mechanism of action. First, cells treated with the drug increased in size and displayed morphological features resembling those of epithelial cells (data not shown). Treated cells presented a significant decrease in *VIM* expression in both control and ABCA1‐overexpressing DLD1 cells (Fig. 7A, left‐hand panels). Together with this, DLD1_ABCA1 cells displayed significantly higher levels of *E‐CAD* (Fig. 7A, right‐hand panels). Thus, RVX‐208 could revert the EMT phenotype present in cells overexpressing the cholesterol transporter. In addition, proliferation rates were slower upon drug treatment in terms of slowed proliferation rates in both DLD1 and Caco‐2 cell lines (Fig. 7B). When cells overexpressing ABCA1 were treated with RVX‐208, ABCA1 protein levels decreased to levels comparable to those in control cells (Fig. 7C). Moreover, RVX‐208 compromised the stability of CAV‐1, causing a reduction in its total protein levels (Fig. 7C). Migration of both DLD1 and Caco‐2 cells was reduced (Fig. 7D). A significant decrease in invasiveness was also observed in DLD1_NoORF, DLD1_ABCA1 and Caco‐2_ABCA1 (Fig. 7D). Thus, the invasive capacities of DLD1_ABCA1 and Caco‐2_ABCA1 returned to normal levels after treatment with the BET inhibitor, indicating putative therapeutic effects of this drugs in CRC patients. Importantly, treatment with RVX‐208 significantly decreased cholesterol transport in both control cells and DLD1_ABCA1 (Fig. 7E). Therefore, the BET inhibitor can target RCT and attenuate malignant features in human CRC‐derived cells. As happened under hAPOA1 treatment, we also observed that *COX‐2* expression was significantly decreased after treatment with RVX‐208 (Fig. 7F). To demonstrate that the rescue of the malignant phenotype was due to APOA1 expression, we used short hairpin RNA. Upon downregulation of *APOA1,* cell proliferation rates were significantly increased in both control (Fig. 8A) and DLD1_ABCA1 cells (Fig. 8B). When cells silenced for *APOA1* and treated with RVX‐208 were interrogated for invasiveness, a significant reversion of the RVX‐208 effect was observed in both DLD1 control cells and in cells overexpressing ABCA1 (Fig. 8C). This demonstrates that the decreased malignancy driven by the BET inhibitor RVX‐208 depends on the up‐regulation of *APOA1* and, in its capacity to regulate ABCA1, CAV‐1 stability and RCT.

## 4 Discussion

The discovery of new biomarkers that would allow the identification and characterization of the initial steps of tumorigenesis, as well as the prediction of disease progression early on, has become of vital importance. Several studies have demonstrated the implication of lipid metabolism regulation in the promotion of migration (Jiang *et al*., 2015), invasion (Fisher *et al*., 2006; Sánchez‐Martínez and Cruz‐Gil, 2015) and angiogenesis, three basic steps of metastasis formation. The results published by Fernández and collaborators together with our previous results concerning the ColoLipidGene signature, support our hypothesis that *ABCA1* could be considered a gene associated with a poor prognosis. We have been able to demonstrate that ABCA1 overexpression leads to EMT and increased invasion. Importantly, equal results were obtained with two cell lines that have been classified inside two distinct consensus molecular subtypes (CMS)‐DLD1 as CMS1 and Caco‐2 as CMS4. These results obtained *in vitro*, are consistent with our study performed with patients, where overexpression of ABCA1 was associated with overall survival independently of the molecular subtype of the cancer (Fernández *et al*., 2017).

Although we did not focus our research on the structure of the plasma membrane, the implications of an increased presence of ABCA1 together with CAV‐1 stabilization on the plasticity of the plasma membrane cannot be ignored. The increased amount of cholesterol in the membrane derived from ABCA1 overexpression could affect the size of raft domains and the composition of the membrane, its fluidity and flexibility, with consequential implications for cell mobility and cell signalling (Guerra *et al*., 2016). According to the data presented here, before diagnosis, ABCA1 can be considered a high‐risk factor that promotes tumour growth and dissemination of the primary lesion.

The BET inhibitors have already been proposed as suppressors of tumorigenesis in several types of cancers (Cheng *et al*., 2013; Puissant *et al*., 2013; Shi *et al*., 2014). Among these kind of inhibitors, apabetalone is the most advanced in its clinical development (Phase III) and is aimed at high‐risk CVD patients with type 2 diabetes mellitus and low HDL. Up to 86% of Phase III clinical trials are considered safe for patients with alimentary, central nervous system, cardiovascular and oncological diseases (Harrison, 2016). As a CVD‐related trial, the probability of apabetalone reaching the next phase is around 55%. Although the trial of apabetalone has not been completed at present, the advanced phase that has not been reached, together with our results, suggest a potential clinical benefit of this drug. Notably, the drug concentration used in our experiments is equivalent to the usage already published for hepatocytes in a study proposing RVX‐208 as treatment for atherosclerotic CVD (Gilham *et al*., 2016). In our two cellular models, apabetalone treatment was equally efficient against invasiveness, indicating that the drug could be efficient in a wide spectrum of patients. One of the main effects of this resveratrol‐derived drug is the increase in *APOA1* transcription. An anti‐tumorigenic activity of this lipoprotein has already been described against melanoma (Zamanian‐Daryoush *et al*., 2013a) as well showing a protective role against the development of a variety of lung diseases, including lung cancer (Gordon *et al*., 2016). Moreover, several studies have shown that APOA1 levels can be considered a biomarker with reduced plasma levels in patients with early stage ovarian cancer compared with normal individuals (Clarke *et al*., 2011; Kim *et al*., 2012; Kozak *et al*., 2003; Moore *et al*., 2006). 2011, 2006, 2012, 2003Lower serum APOA1 levels were also associated with higher risk of breast cancer as well as breast cancer recurrence (Chang *et al*., 2007) (Lane *et al*., 1995). The exact mechanism by which APOA1 can modulate tumour malignancy is not yet fully understood. One of the hypotheses is that APOA1 is implicated in the biology of the tumour microenvironment (Zamanian‐Daryoush *et al*., 2013). Consistent with our results demonstrating that hAPOA1 treatment attenuates ABCA1‐driven phenotypes, we also showed that, through *APOA1* up‐regulation, RVX‐208 diminishes cell malignancy in CRC‐derived cells. We propose here that the imbalance in the expression of ABCA1 and APOA1 is responsible for the malignant features deriving from ABCA1 overexpression. The fact that the high basal levels of ABCA1 in Caco‐2, a cell line with low rates of proliferation and low invasive capacities, are accompanied by high levels of APOA1 supports our hypothesis that disturbing this balance by a further increase of ABCA1 leads to increased proliferation and invasiveness. As APOA1 could be easily detected in human plasma, the increased concentration of the protein in plasma extracted from CRC patients could be used as marker of treatment efficacy. RVX‐208 treatment modulates ABCA1 expression, regulates CAV‐1 stability and controls RCT.

Further efforts and resources should be directed towards acquiring knowledge of cholesterol balance and cancer progression. Both cell autonomous effects and their role in the tumour microenvironment may be crucial to fully understand the importance of cholesterol metabolism in cancer prognosis.

## 5 Conclusions

The expression levels of the cholesterol transporter ABCA1 can be considered new prognosis markers for colorectal cancer malignancy. ABCA1 overexpression leads to EMT, and promotes migration and increases invasive capacities by regulating CAV‐1 stability. By modulating ABCA1 expression and cholesterol processing, APOA1 is able to reverse the malignant phenotypes displayed by cells overexpressing the RCT regulator. Thus, intracellular cholesterol metabolism and the apolipoprotein APOA1 emerge as new relevant players in CRC progression to metastasis. Based on these results, we propose here, for the first time, the potential use of apabetalone, a stimulator of *APOA1* so far only considered for CVD, as a bona fide inhibitor of colorectal cancer invasiveness.

## Acknowledgements

We thank Susana Molina for technical support in the management of SII‐CRC and SIII‐CRC patients. This work has been supported by Ministerio de Economía y Competitividad del Gobierno de España (MINECO, Plan Nacional I+D+i AGL2016‐76736‐C3), Gobierno regional de la Comunidad de Madrid (P2013/ABI‐2728, ALIBIRD‐CM), EU Structural Funds and AMAROUT‐Marie Curie actions (COFUND2014‐51539‐04).

## Author contributions

CAP designed the experiments, collected the data, performed the analyses and wrote the manuscript. JF provided human samples from patients. ARM and GR provided financial support, critical review and final approval of the manuscript.

## Supporting information


**Fig. S1.** ABCA1 overexpression promotes epithelial‐to‐mesenchymal transition and leads to increased invasiveness.

**Fig S2.** ABCA1 overexpression favors three‐dimensional growth and promotes invasion in spheroids embedded in Matrigel™.

**Fig. S3.** Increased invasiveness of CRC cells overexpressing ABCA1 is dependent on caveolin‐1 regulation.

**Fig. S4** Exogenous human recombinant APOA1 diminishes proliferation of Caco‐2_ABCA1 cells to levels comparable to those in controls.

**Table S1.** Statistical analysis for migrating and invading properties in DLD1 cells.
