# Supplementary material for: ABCA1 overexpression worsens colorectal cancer prognosis by facilitating tumour growth and caveolin‐1‐dependent invasiveness, and these effects can be ameliorated using the BET inhibitor apabetalone
Source: Mol Oncol. 2018 Sep 17;12(10):1735–52. doi: 10.1002/1878-0261.12367 (PMC6166002; doi:10.1002/1878-0261.12367)
Supplement: Supplementary file 1 — Fig. S1. ABCA1 overexpression promotes epithelial‐to‐mesenchymal transition and leads to increased invasiveness. [file MOL2-12-1735-s001.pdf]

Supplementary Figure 1:

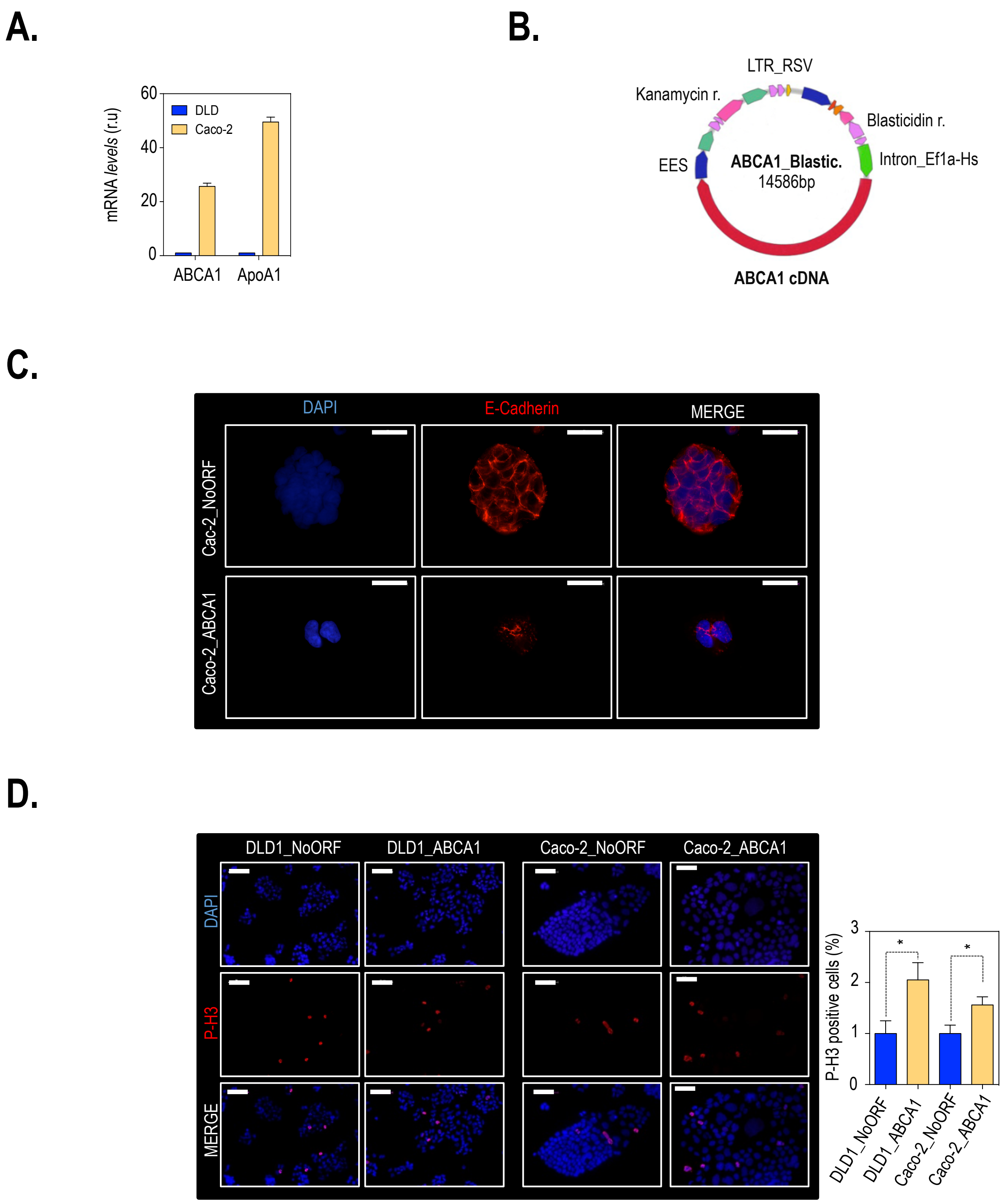

Supplementary Figure 1: A) Basal mRNA levels of ABCA1 and ApoA1 in DLD1 and Caco-2 cell lines. The quantitative RT-PCR was performed by triplicate. Each column represents the mean±SEM. (ABCA1: 1,000 ± 1,502e-005; ApoA1: 25,70 ± 0,8461). B) Plasmid backbone (ABCA1_Blasticidin) used for the generation of two different epithelial colorectal adenocarcinoma-derived stable cell lines (DLD1 and Caco-2) for ABCA1 overexpression. LTR=Long terminal repeats. RVS=Rous sarcoma virus. EES=Exonic splicing enhancer. r=resistance. C) Localization of E-cadherin in Caco-2 cells, both control and ABCA1 overexpressing cells are shown. Nuclei are stained with DAPI and are shown in blue. E-cadherin is shown in red. mRNA levels of E-cadherin and Vimentin are displayed in the left panel. Scale bar corresponds to 20µm. D) Phospho-Histone H3 immunostaining in DLD1 and Caco-2 cells. Nuclei are stained with DAPI and are shown in blue. Phospho-Histone H3 is shown in red, microtubules are shown in green by anti a-tubulin immunodetection. Scale bar corresponds to 50µm. The quantification is represented on the histogram on the right. n=3; 10 pictures per condition were taken and quantified. Each column represents the mean±SEM. DLD1_NoORF: 1,000 ± 0,2505; DLD1_ABCA1:2,052 ± 0,3368 p-value=0.0187 Caco-2_NooRF: 1,000 ± 0,1659, Caco-2_ABCA: 1,561 ± 0,1593, p-value=0.0296). Significance between groups was determined by t-test. All reported p values were two-sided.
