# Supplementary material for: ABCA1 overexpression worsens colorectal cancer prognosis by facilitating tumour growth and caveolin‐1‐dependent invasiveness, and these effects can be ameliorated using the BET inhibitor apabetalone
Source: Mol Oncol. 2018 Sep 17;12(10):1735–52. doi: 10.1002/1878-0261.12367 (PMC6166002; doi:10.1002/1878-0261.12367)
Supplement: Supplementary file 2 — Fig S2. ABCA1 overexpression favors three‐dimensional growth and promotes invasion in spheroids embedded in Matrigel™. [file MOL2-12-1735-s002.pdf]

Supplementary Figure 2:

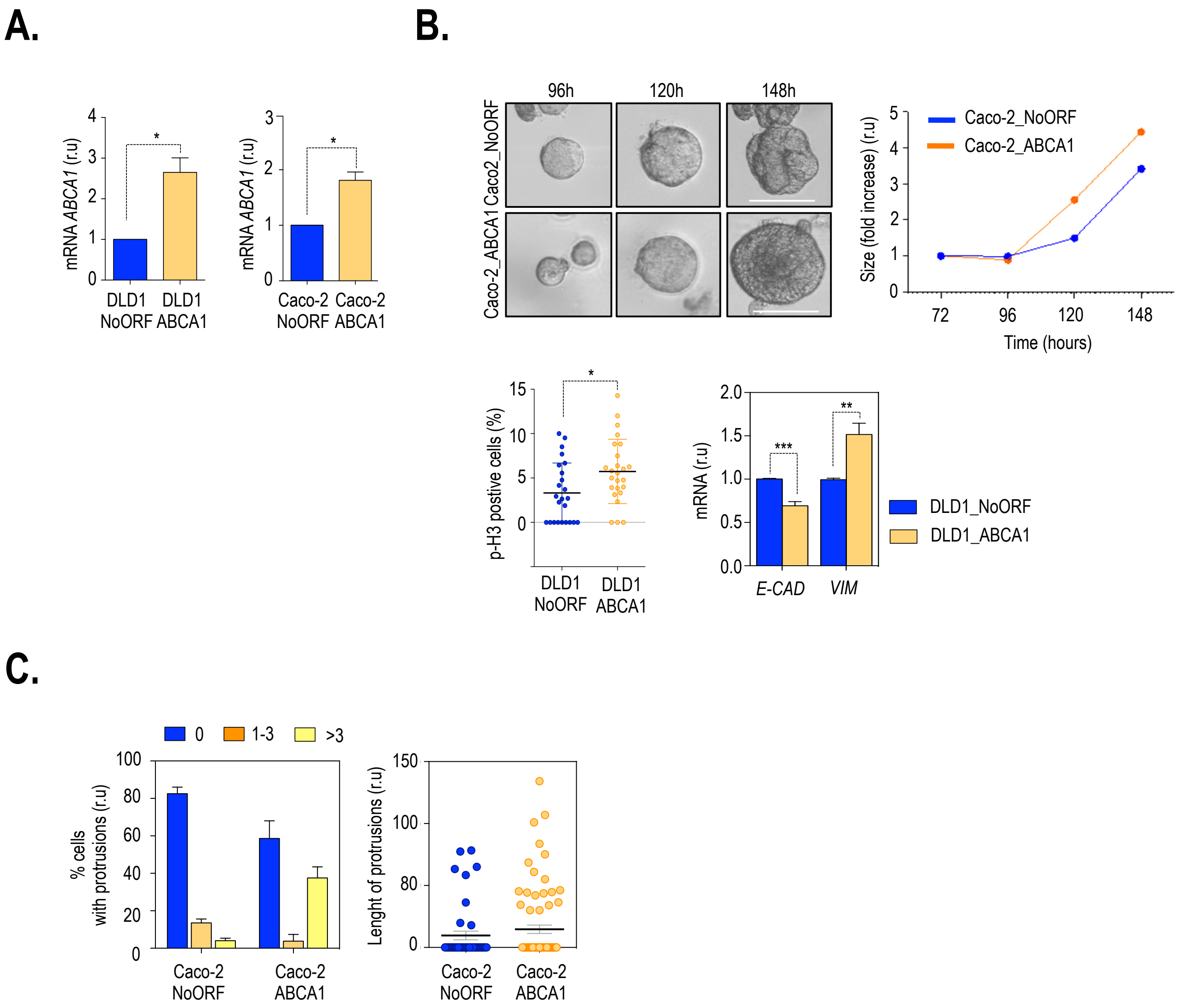


**Supplementary Figure 2:** A) ABCA1 mRNA levels of expression in spheroids derived from DLD1 and Caco-2 cell lines. The quantitative RT-PCR was performed in triplicate. Each column represents the mean±SEM. (DLD1_NoORF: 1,000 ± 3,576e-007, DLD1_ABCA1: 2,649 ± 0,3553, p-value=0.0369; Caco-2_NoORF: 1,000 ± 2,146e-006; Caco-2_ABCA1: 1,810 ± 0,1524, p-value=0.0259). B) Monitorization of cell growth in a spheroid formation assay in basement membrane matrixes (Matrigel). Growth of Caco-2 spheroids are represented in the panel on the left. The graph represents the average of thirty different spheres. Scale bar corresponds to 50µm. The histograms represent quantification of Histone H3 phosphorylation (DLD1_NoORF: 3,311 ± 0,6882; DLD1_ABCA1: 5,736 ± 0,7247, p-value=0.0193) and the levels of mRNA of E-cadherin (E-Cad. DLD1_NoORF: 1,003 ± 0,003161; DLD1_ABCA1: 0,6925 ± 0,02835, p-value=0.0004) and Vimentin (Vim. DLD1_NoORF: 0,9942 ± 0,009214; DLD1_ABCA1: 1,515 ± 0,07528, p-value=0.0024). Each column or dot plot graph represents the mean±SEM. n=3; 10 pictures per condition were taken and quantified. C) Percentage of spheroids with protrusions are represented. The significance was determine by ANOVA analysis ($F_{(2, 6)}$ = 34,75; p= 0.0005). In the dot plot graph, values corresponding to the longest protrusions per spheroid are quantified. Ten to twenty spheres were analyzed in each of the three experimental replicates. Each column or dot plot graph represents the mean±SEM. (Caco-2_NoORF: 9,694 ± 3,474; Caco-2_ABCA1: 14,69 ± 3,368, p-value=0.3370). Significance between groups was determined by t-test. All reported p values were two-sided.
