# Supplementary material for: ABCA1 overexpression worsens colorectal cancer prognosis by facilitating tumour growth and caveolin‐1‐dependent invasiveness, and these effects can be ameliorated using the BET inhibitor apabetalone
Source: Mol Oncol. 2018 Sep 17;12(10):1735–52. doi: 10.1002/1878-0261.12367 (PMC6166002; doi:10.1002/1878-0261.12367)
Supplement: Supplementary file 3 — Fig. S3. Increased invasiveness of CRC cells overexpressing ABCA1 is dependent on caveolin‐1 regulation. [file MOL2-12-1735-s003.pdf]

Supplementary Figure 3:

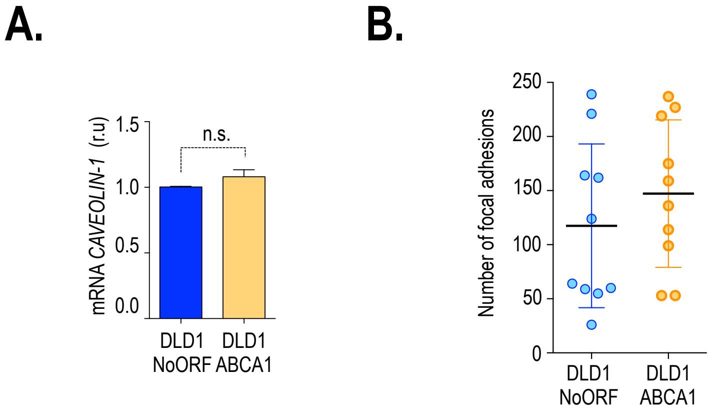

Supplementary Figure 3: A) mRNA Caveolin-1 levels of expression in both DLD1 control and ABCA1 overexpressing cells. n=3. Each column represents the mean±SEM. DLD1_NoORF: 1,003 ± 0,002932; DLD1_ABCA1: 1,080 ± 0,03099. p-value=0.0676. B) Plot showing the quantification of the number of focal adhesions. The experiment was performed by triplicate. Each dot plot set of values represent the mean±SEM. DLD1_NoORF:117,4 ± 23,93; DLD1_ABCA1: 147,2 ± 21,55, p value=0.3660. Significance between groups was determined by t-test. All reported p values were two-sided.
