# Supplementary material for: ABCA1 overexpression worsens colorectal cancer prognosis by facilitating tumour growth and caveolin‐1‐dependent invasiveness, and these effects can be ameliorated using the BET inhibitor apabetalone
Source: Mol Oncol. 2018 Sep 17;12(10):1735–52. doi: 10.1002/1878-0261.12367 (PMC6166002; doi:10.1002/1878-0261.12367)
Supplement: Supplementary file 4 — Fig. S4 Exogenous human recombinant APOA1 diminishes proliferation of Caco‐2_ABCA1 cells to levels comparable to those in controls. [file MOL2-12-1735-s004.pdf]

Supplementary Figure 4:

**A.**

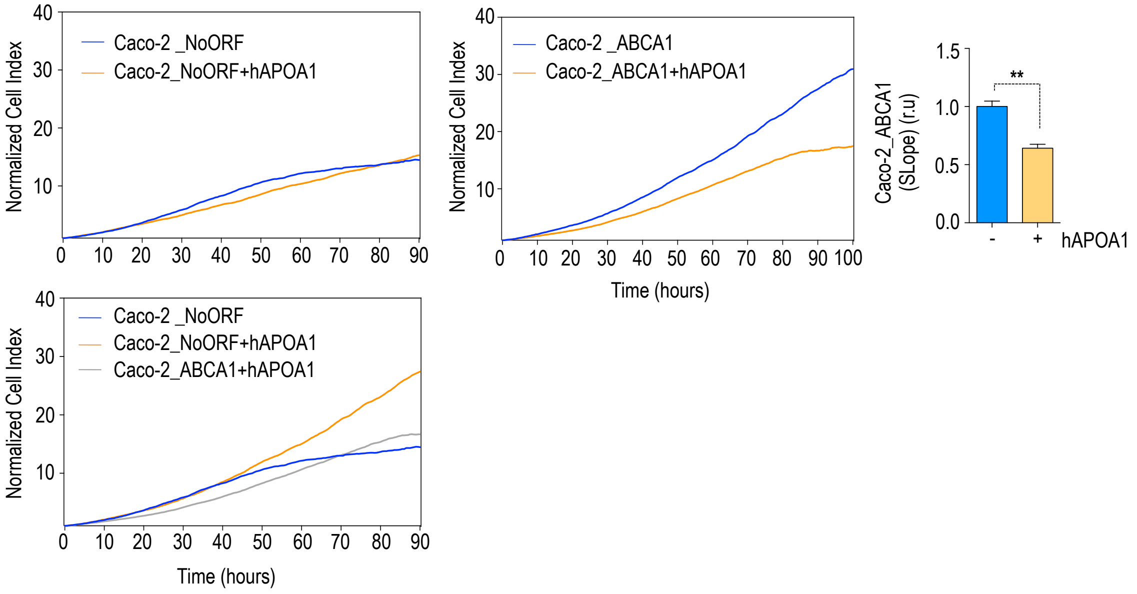


**Supplementary Figure 4:** A) Growth curves for Caco-2 cells treated with human recombinant ApoA1. Slope for Caco-2_ABCA1 is shown in right panel. Each column represents the mean±SEM of three independent experiments. Caco-2_NoORF: 1,000 ± 0,04746; Caco-2_ABCA1: 0,6414 ± 0,03464, p-value=0.0036). Significance between groups was determined by t-test. All reported p values were two-sided.
