# Supplementary material for: ABCA1 overexpression worsens colorectal cancer prognosis by facilitating tumour growth and caveolin‐1‐dependent invasiveness, and these effects can be ameliorated using the BET inhibitor apabetalone
Source: Mol Oncol. 2018 Sep 17;12(10):1735–52. doi: 10.1002/1878-0261.12367 (PMC6166002; doi:10.1002/1878-0261.12367)
Supplement: Supplementary file 5 — Table S1. Statistical analysis for migrating and invading properties in DLD1 cells. [file MOL2-12-1735-s005.pdf]

Supplementary Table I:

		DLD1_ApoA1				DLD1_Double			
	DLD1_NoORF	Migration		Invasion		Migration		Invasion	
	Mean±SEM	Mean±SEM	p value	Mean±SEM	p value	Mean±SEM	p value	Mean±SEM	p value
**Migration**	0,9601±2278	2,833±0,8038	0,02701 (*)			3,758±1,276	0,0182 (*)		
**Invasion**	0,7999±0,1320			0,3158±0,1558	0,0218 (*)			0,9257±0,302	0,6652
	**DLD1_ABCA1**	**Migration**		**Invasion**		**Migration**		**Invasion**	
	Mean±SEM	Mean±SEM	p value	Mean±SEM	p value	Mean±SEM	p value	Mean±SEM	p value
**Migration**	6,116±1,143	2,833±0,8038	0,0224 (*)			6,116±1,143	0,1759		
**Invasion**	1,568±0,1718			0,3158±0,1558	<0,0001 (****)			0,9257±0,302	0,0533

Significance between groups was determined by t-test. All reported p values were two-sided.

Statistical analysis for migrating and invading properties
